# Inferior Vena Cava Filter-Induced Thrombosis: Filter Insertion Prior to Catheter-Directed Thrombolysis and Successful Double-Filter Retrieval after Prolonged Indwelling Time

**DOI:** 10.3400/avd.cr.20-00076

**Published:** 2020-12-25

**Authors:** Wakako Fukuda, Takashi Shibuya, Kenichi Watanabe, Masato Ohno, Tomoaki Kudo, Ikuo Fukuda, Mitsunori Kaneko

**Affiliations:** 1 Department of Cardiovascular Surgery, Suita Tokushukai Hospital, Suita, Osaka, Japan; 2 Department of Cardiovascular Surgery, Osaka University Graduate School of Medicine, Suita, Osaka, Japan

**Keywords:** IVC filter, prolonged dwell time, late complication

## Abstract

Extended inferior vena cava (IVC) filter implantation time increases the risk of complications in patients. Here we present the case of a 72-year-old woman with IVC filter-induced thrombosis who underwent catheter-directed thrombolysis with prophylactic IVC filter placement. Two IVC filters were successfully retrieved 70 and 1858 days post placement. The decision to insert an IVC filter should be carefully considered with appropriate indications and all filters should be removed after the risk of deep vein thrombosis has resolved.

## Introduction

Venous thromboembolism (VTE) is a leading cause of morbidity and mortality.^1)^ The role of anticoagulants in the treatment of VTE is well established. An inferior vena cava (IVC) filter placement must be considered when anticoagulation is contraindicated or ineffective, or when anticoagulation fails. Three types of IVC filters are available in Japan: permanent, temporal, and retrievable. Retrievable filters can be retrieved or left in place after temporary risk of pulmonary embolism (PE) or the contraindication to anticoagulation has resolved.^2)^ Although designed to be removed, the majority of filters are left in place for a prolonged period of time, and studies have demonstrated that a prolonged indwelling time results in device-related complications.^3)^ In this study, we report a case of IVC filter-induced thrombosis. A second filter was inserted prior to catheter-directed thrombolysis (CDT), and both filters were successfully removed 70 and 1858 days post placement.

Written informed consent was obtained from the patient for publication of this case report and accompanying images.

## Case Report

**Figure 1:**
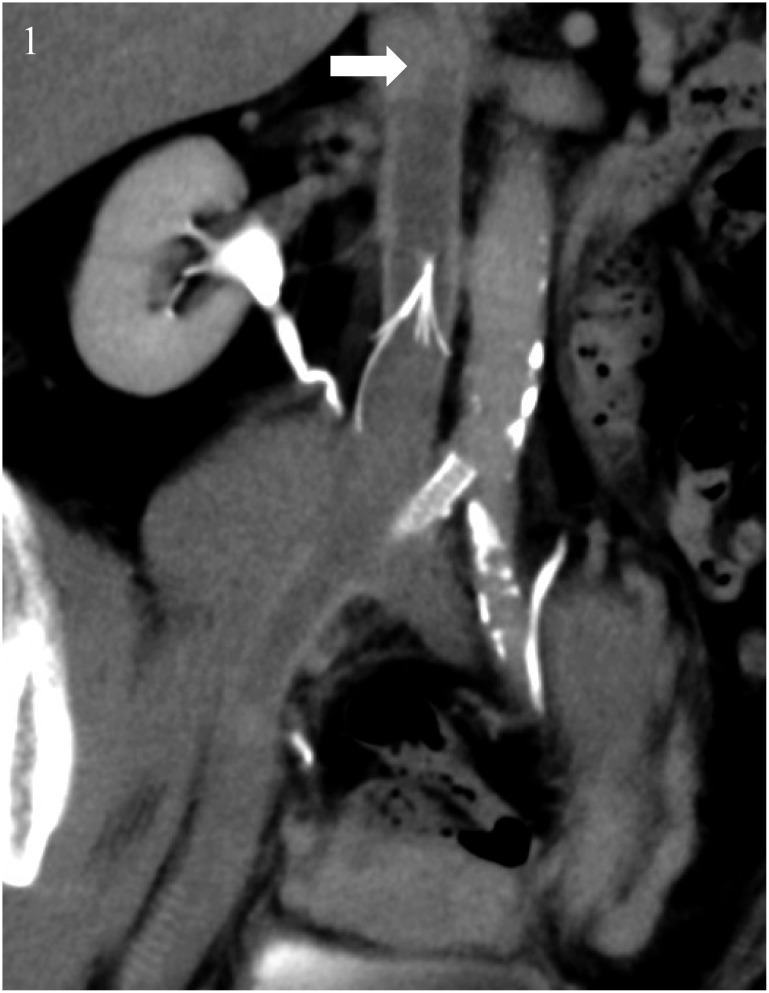
Fig. 1 Coronal image demonstrating extensive thrombus of both iliac veins with thrombus extension to the inferior vena cava, the previously deployed infrarenal inferior vena cava filter, and beyond. The top of the IVC thrombus is at the left renal vein (white arrow).

**Figure 2:**
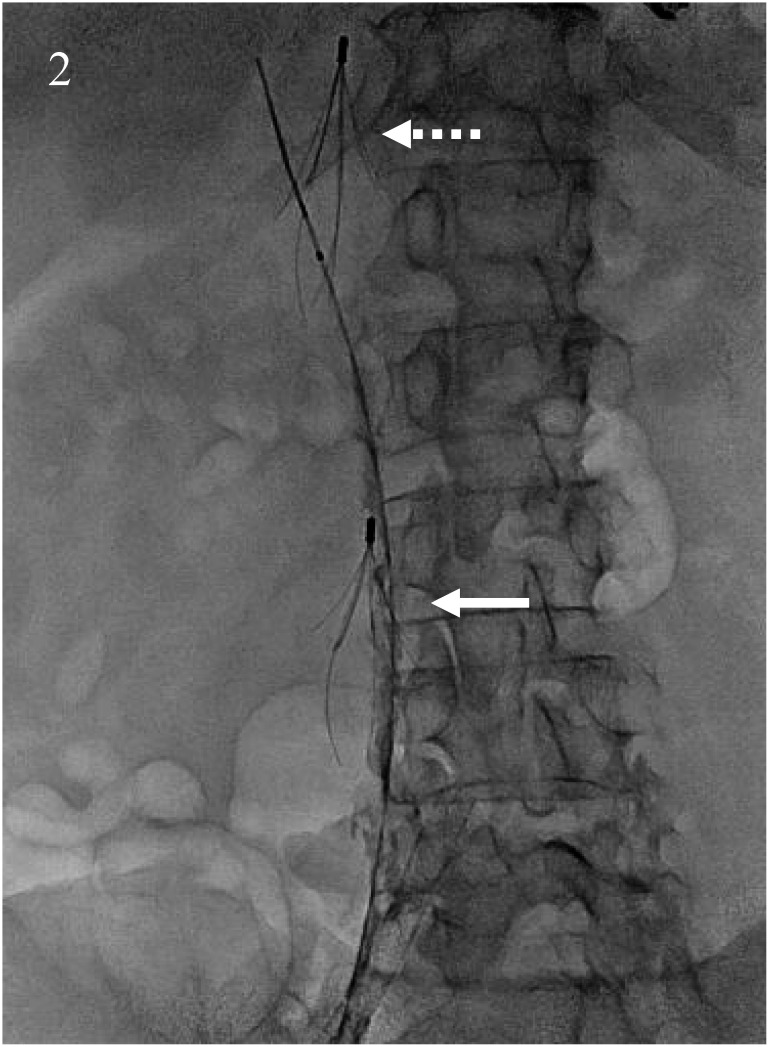
Fig. 2 Posteroanterior radiograph demonstrating the distal (white arrow), proximal (dashed arrow) inferior vena cava filters, and catheter-directed thrombosis catheter.

**Figure 3:**
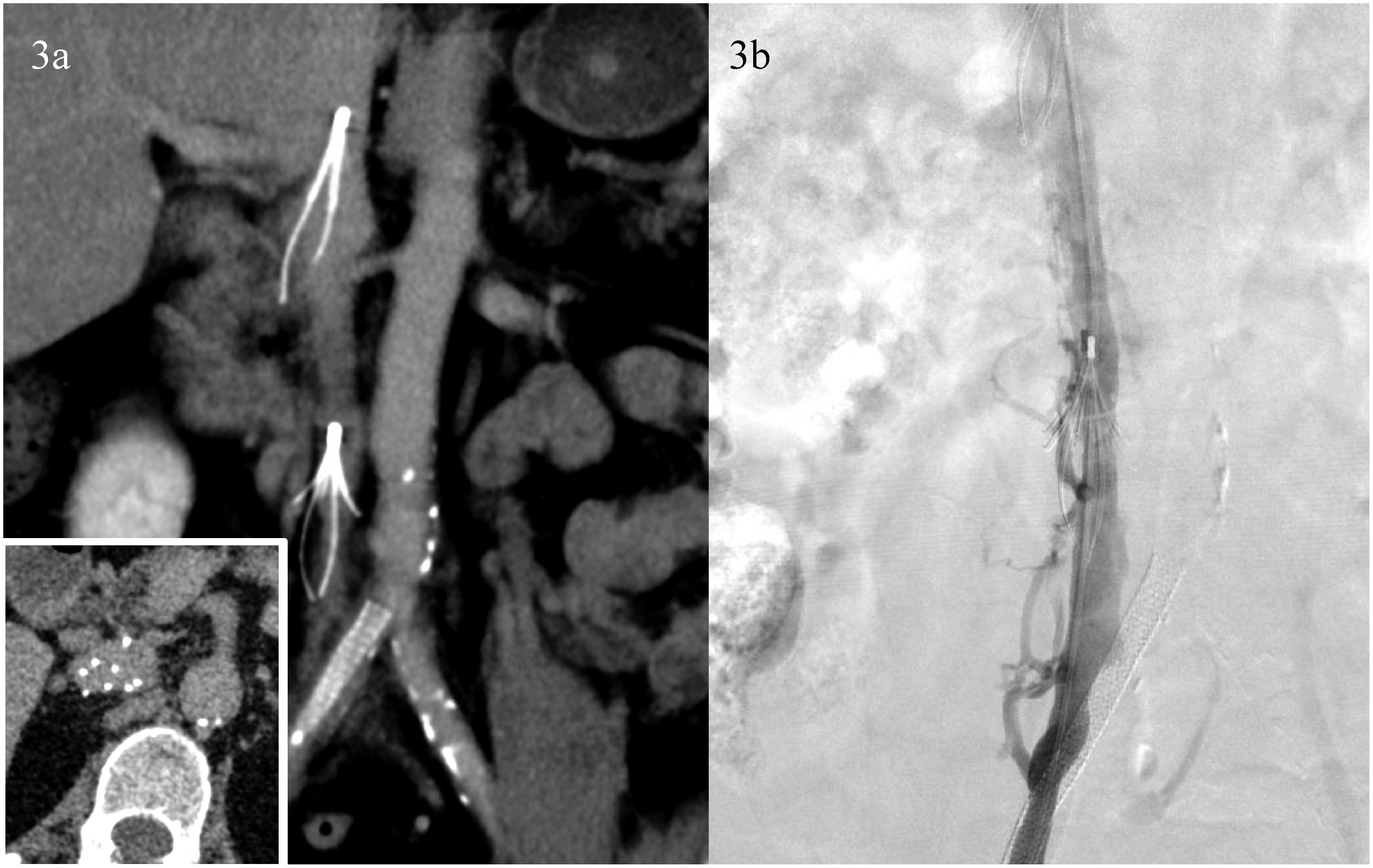
Fig. 3 Computed tomography (**a**) and angiogram (**b**) taken prior to inferior vena cava (IVC) filter retrieval show no thrombus in the IVC. Axial computed tomography image (**a**, lower left corner) demonstrates proximal filter penetration. However, no organs or the structure are involved.

A 72-year-old woman with a history of arteriosclerosis obliterans, abdominal wall hernia, and deep vein thrombosis (DVT) had a retrievable IVC filter (ALN implants Chirurgicaux®, Ghisonaccia, France) placed at an outside facility for perioperative mechanical prophylaxis in 2015. The filter was never retrieved. At 5 years of postfilter insertion, she was referred to our hospital with swollen right leg. Doppler ultrasound of the lower limbs confirmed an iliofemoral DVT. A contrast-enhanced computed tomography (CT) scan revealed a thrombus involving the right small saphenous vein, popliteal vein, femoral vein, and both iliac veins with extension to the infrarenal IVC, the IVC filter, and beyond (**Fig. 1**). Workups for acquired and inherited thrombophilia were negative. CDT of the DVT was planned after suprarenal IVC filter placement. An ALN IVC filter was deployed between the left renal vein and the hepatic vein. The patient was placed in the prone position and CDT was performed using the right small saphenous vein approach. A 4Fr infusion catheter (Fountain® Infusion Systems, Merit Medical Systems Inc., UT, USA) was placed with the tip embedded in the proximal end of the thrombus in the IVC (**Fig. 2**). Urokinase was first injected as a bolus dose of 120,000 U followed by the continuous infusion of 240,000 U/day from the infusion catheter. Heparin was also administered at 15,000 U/day with a target of a 1.5–2.5-fold activated partial thromboplastin time. Surveillance venography was performed every 24–48 h to assess the thrombolysis degree and adjust the catheter position to facilitate thrombolysis. After 8 days of CDT, venography revealed that almost all the thrombi were dissolved. A total dose of 1,080,000 U of urokinase was administered. Her symptoms improved, and she was discharged on the oral anticoagulant rivaroxaban on the 14th day. Follow-up ultrasonography revealed no DVT and CT showed no trapped thrombus in either IVC filter. Upon the patient providing informed consent, the two filters were retrieved after implantation periods of 70 days (proximal filter) and 1858 days (distal filter) through the right internal jugular vein approach. Enhanced CT showed no thrombus in IVC (**Fig. 3a**). IVC cavography also demonstrated that the IVC was patent without a thrombus (**Fig. 3b**). There was no filter tilt. Using a 9 Fr extraction device, the two filters were successfully removed. The final cavography showed no evidence of active extravasation of contrast medium. She was admitted for 24-hour observation and discharged thereafter. Follow-up ultrasonography performed at 1, 3, and 6 months and 1 year showed a patent IVC with regular flow. The DVT did not recur.

## Discussion

The use of IVC filters has increased since the approval of retrievable filters, and the availability of retrievable filters has changed their practice patterns. As new devices have become available, filter placement indication has expanded. While there is general agreement with respect to classic indications, guidelines on expanded indications are missing given the lack of prospective studies. The rationale for retrievable filter use is, for the most part, the result of a single prospective randomized trial. The PREPIC (Prevention du Risque d’Embolie Pulmonaire par Interruption Cave) study’s initial 2-year results suggested that the IVC filter provided significant additional short-term protection against PE than anticoagulation.^4)^ However, at 8 years, the IVC filter reduced the risk of PE while increasing the incidence of DVT and had no effect on survival.^5)^ Based on these results, many clinicians began questioning the prolonged placement of IVC filters.

In our case, the first IVC filter was implanted prophylactically prior to an abdominal hernia operation at an outside facility because the patient had a history of DVT and the clinicians considered her at high risk of developing perioperative DVT. The filter was left in place for over 4 years without anticoagulation therapy, possibly putting her at increased risk for thrombosis. There are no randomized trials to support the use of prophylactic IVC filters for any group of patients.

Many retrievable filters are not retrieved for various reasons. Only a small percentage of retrievable IVC filters (20%–50%) are reportedly removed.^6)^ Reasons for filter retrieval failure include lack of patient follow-up, large thrombus within the filter, significant filter tilt, embedded filter tip, embedded filter struts, strut perforation, and filter fracture.^7)^ Physician oversight is another reason for retrieval failure.^6)^

Prolonged indwelling time has been associated with device-related complications and a significantly increased risk of complicated retrieval.^8)^ After prolonged indwelling time, as many as 40%–60% of retrievable filters cannot be removed using standard techniques because of the resulting filter adhesion to the wall, filter tilt or filter malposition.^7)^ Although the first filter in our patient remained in place for 1858 days, we were able to easily remove both filters using the conventional technique because our patient had no filter-related complications. The design of the ALN filter might also have attributed to the uneventful filter retrieval. A conical ALN filter has six shorter anchoring struts with hooks and three longer centering struts; all struts have differing lengths. Mismetti et al. reported on ALN filter use in a cohort of 220 patients, wherein filter removal was attempted in 25.3% of patients with success rates of 92.7% for one attempt and 100% for two attempts. The median indwelling time was 166 days.^9)^ In another study examining early and late removal of 123 ALN filters, removal was successful in 99% patients, with a mean dwell time of 93 days (46% of filters were retrieved at >2 months).^10)^ In a retrospective study comprising 29 patients whose ALN filters had been in place for more than 1 year (mean dwell, 25.6 months; range, 14.8–40.8 months), the retrieval success rate was 100%.^8)^ Although excellent outcomes of the ALN filters have been reported, certain situations may make retrieval challenging, and, in such cases, advanced retrieval techniques may be necessary. Vascular surgeons and radiologists involved in IVC filter insertion should have the necessary knowledge and skills in various filter retrieval techniques. The retrieval of IVC filters after a long indwelling time can be technically difficult; however, it should be attempted to prevent complications including caval thrombus, recurrent DVT, filter penetration, and migration.

## Conclusion

IVC filter-induced thrombosis was treated with CDT after additional placement of a filter, and both filters were successfully removed 70 and 1858 days post placement. A prolonged indwelling time of an IVC filter increases the risk of complications. Clinicians should consider the benefits and risks for each patient, and the decision to insert a filter should be carefully considered with appropriate indications. A standardized monitoring and follow-up plan should be initiated with the assessment of filter retrieval eligibility. Finally, filter removal should be planned in all patients for whom the requirement for an IVC filter is no longer indicated.
